# Gastric Ulcer

**Published:** 1891-02-28

**Authors:** 


### THE PRACTITIONER'S MIRROR AND RETROSPECT.

[*The Editor will be glad to receive offers of co-operation and contributions from members of the profession. All letters should be addressed to* THE EDITOR, THE LODGE, PORCHESTER SQUARE, LONDON, W.]

### MEDICINE.

### GASTRIC ULCER.

There are few complaints which occasion more anxiety, both in diagnosis and treatment, than ulcer of the stomach, and this in spite of all the labour which has been expended in finding a scientific groundwork for both. There may be few or no symptoms before a sudden and fatal hæmorrhage occurs; or there may be typical symptoms lasting for a long period, about which it is impossible to say afterwards whether they have been really produced by gastric ulcer at all; and there are cases which exhaust the perseverance of both physician and patient, and which, after apparently recovering under rigorous diet, recur time after time when the patient returns to ordinary life. The varying frequency of gastric ulcer in different countries has long been noted and recently Von Sohlern has shown cause to refer this to difference in diet rather than to climate or race. The populations of the Bavarian Alps, the Rhön, and of Greater Russia possess a singular immunity. Only ·2 per cent. of cases occur among them, as against 4 or 5 per cent. in the rest of Europe. Here are races and climates of wide diversity, yet the populations, amounting to 25,000,000, show a remarkable uniformity in their immunity from the disease. Sohlern traces this to the large amount of potash in their diets, which exceeds that found in the average German diet in the proportion of 10 to 7 5. The superior alkalinity of the blood, he thinks, affords an especial protection from the corrosive action of the gastric juice, and as the red blood corpuscles act as the chief carriers of potash, he would look to diseases which produce a change or lessening of these corpuscles as predisposing to gastric ulcer. The alkalinity may be so great that such diseases may have little effect. Hence chlorosis, though common enough in these districts, rarely leads to ulcers. His view, if not altogether proved, has considerable weight, but it would be interesting to discover where it is borne out by similar results among the great vegetarian populations of the East, if indeed their potash consumption is not reduced by special circumstances. In England we are accustomed to regard excessive tea drinking as one of the predisposing causes, but if we notice the greater prevalence of the disease in Germany, where tea is little used, and its rarity in Greater Russia, where tea is universal, the accusation would seem to have little importance. Openchowski has investigated a form of degenerative softening of the vessel walls which he finds in cases of ulcer, and which probably renders them unable to carry on their functions when exposed to the varying pressure of the muscular wall of the stomach. Unless some such change can be proved to be common, it is not altogether easy to define what conditions lead to the interruption of the circulation in certain circumscribed portions of the stomach wall, which was the immediate cause of the disease postulated by Virchow.

No doubt when stasis does occur erosion follows quickly enough, but even then in the normal state healing succeeds just as rapidly. The advocates of a nerve influence have certain facts in their favour. Talma has recently produced ordinary round ulcers by stimulating the left vagus for some hours, and thus inducing a violent contraction of the wall, and Byron Bramwell notes the increase of the epigastric reflex on the right side when ulcers are present, though this is more likely to be the effect of the irritation they cause. On the other hand, Schtscherbakou has recently produced typical ulcers by injecting an irritant into the gastric vessels, but they healed quickly without leaving a trace behind. If, however, the acidity of the stomach was raised they increased in size and went on to perforation. This was also the case when a changed state of the blood was produced by bleeding, or by the introduction of substances such as anilin, which lessened its functional activity. The probability is that incipient ulceration is common, and possibly local interruption of the circulation may occur not infrequently, but the difficulty is to ascertain under what conditions the ulcer tends to spread. Embolism, venous thrombosis, spasmodic contraction of the muscular coat and vasomotor changes have all been invoked as producers of the circulatory derangement, but as Ziemsen points out, there are grave difficulties in the way of the first explanation in view of the free anastomoses of the arteries. It is more probable that small portions of irritating and undigested food adhere to a given spot, calling forth on the one hand local spasmodic contractions of the muscular coat beneath, and on the other stirring up the cells to pour out excessive amounts of acid which react on the constricted spot. Nor is this all. Fermentative changes follow, other acids are produced, and micro-organisms, protected by the adherent mass, invade the tissue, which, thus attacked from front and rear, soon becomes necrotic. All this, however, hardly explains why healing does not take place as in wounds and surgical operations. The light thrown on the different varieties of acid dyspepsia by recent investigations may lead to the elucidation of this problem. As Professor Hamilton pointed out, there are two distinct groups of these affections, the one owing to a deficiency of hydrochloric acid, with excess of lactic and other organic acids, and the other to an increase and irregular secretion of hydrochloric. As to the latter, Riegel states that it may continue for years without notable symptoms, but that it predisposes to ulcer. The acid may be poured out in excess, not only on the introduction of food, but also during fasting, so that the digestion of starchy food is hindered and tends to accumulate in the stomach. The condition is apt to occur in certain nerve diseases as well as independently of any known cause, but, on the whole, it seems to be rarer than the acidity due to an excess of organic acids. There is reason to think that this is, at any rate, one of the conditions or abnormal states in which ulcers tend to spread and perforate. From the experiments we have mentioned, as well as from the older ones of Pavy, it seems clear that there must be usually both an alteration or interruption of the circulation, together with increased acidity, before the powers of repair can be overcome. Another way in which this result is at times reached is through the action of certain infective microbes. Böllcher demonstrated their presence in certain cases long ago, but we have little definite information about their action. In connexion with this point we may notice the frequent occurrence of ulcer in endocarditis, tubercle, trichinosis, and chlorosis. An invasion of pathogenic microbes may of itself cause ulcers or interfere with healing just as in external wounds and ulcers, and lead to rapid perforation. Probably the round ulcers formed in chlorosis in young women are often caused in this way. The question as to what microbes can live in the stomach has been much debated. Kurlow and Wagner, while insisting on the destructive action of healthy juice on many germs, confess that there are certain forms normally present there, and others, such as the staphylococci and the spores of anthrax, which can survive the effect of the gastric fluid. Dr. William Hunter made a report of very great interest before the Pathological Society, on the 20th ult., as to a case of sub-acute gastritis, which, for three years, had presented the symptoms of pernicious anæmia, without any sign of dyspepsia except a raw tongue. Dr. Hunter traced the blood changes to the presence of two products of bacterial activity, putrescine and cadaverine, which he found in the urine, and these alkaloids to the action of large masses of bacilli, which he demonstrated in the veins and lymphatics of the ulcerated parts of the stomach.

The formation of gastric and duodenal ulcers after severe burns has been explained as the result of vasomotor inhibition; but it is probably only a case of embolism resulting from pyæmic infection through the raw surfaces.

Dr. Ord has classified gastric ulcers in two groups, the first comprising the deep, round ulcers common in young women. Here pain is a more prominent symptom than vomiting; but in uncomplicated cases there may be pain only after meals when it follows the introduction of food, sooner or later, as the ulcer is situated nearer or farther from the cardiac end. He considers that the insatiable hunger sometimes seen is caused by adhesions which prevent the walls of the stomach coming together as they normally do when the organ is empty. The second class is made to include the shallow, extensive ulcers, found in persons of middle age and either sex. The patients are wasted and have little pain, but frequently vomit an extremely acid fluid, more or less mixed with blood. No doubt these represent two distinct types, but whether the difference depends on anything more than the age and habits of the sufferers remains to be proved. It appears from Brinton's statistics that the frequency of ulcer in women is twice that of the same affection occurring in men. Yet it is by no means rare in certain classes of workmen, such as barmen and cooks. Very noteworthy, too, is its frequency in shoemakers, who press the last against their chests and habitually suffer from dyspepsia.

The difficulties of diagnosis are so great that we are often obliged to fall back on Ziemsen's rule of treating as gastric ulcer all doubtful cases. Hysteria cancer, aneurism, gastralgia, may present symptoms for a long time quite undistinguishable. In a case where severe hæmorrhage from a gastric ulcer suddenly occurred, a strongly pulsating abdominal aorta added greatly to the difficulty of diagnosis, and alarmed the patient almost as much as the hæmorrhage. The tenderness of the lowest dorsal spines noted by Cruveilhier does not seem to be frequent. If dyspepsia is present it is important to determine its nature exactly, the more so since ulcer appears to occur chiefly in certain varieties of this affection. Dr. Anderson has rightly insisted on the great importance of complete rest in bed as the first element of treatment, both on account of the greater facility with which healing takes place, and the smaller danger of perforation. He appears to have found excellent results, too, from the administration of the artificial Carlsbad salts (which increases the alkalicity of the blood) in combination with the usual very small, repeated meals of peptonised or at least fluid food. Certain cases must be treated by rectal alimentation, but as soon as possible attention must be directed to the improvement of the general health, and especially to the chlorosis, dyspepsia, and other conditions which rendered the formation and growth of the ulcer possible. If this cannot be done the probability of a relapse must be faced.

